# Deciphering Morphological Variability: Addressing Taxonomic Ambiguities in Contemporary Species Delimitation (Hymenoptera, Figitidae)

**DOI:** 10.3390/insects17010054

**Published:** 2026-01-01

**Authors:** Mar Ferrer-Suay, George E. Heimpel, Ehsan Rakhshani, Jesús Selfa

**Affiliations:** 1 Departament de Zoologia, Facultat de Ciències Biològiques, Campus de Burjassot-Paterna, Universitat de València, Dr. Moliner 50, Burjassot, E-46100 València, Spain; 2 Department of Entomology, University of Minnesota, St. Paul, MN 55108, USA; 3 Department of Plant Protection, College of Agriculture, University of Zabol, Zabol P.O. Box 98615-538, Iran

**Keywords:** *Alloxysta*, Charipinae, *Phaenoglyphis*, phylogeny, taxonomy

## Abstract

Species delimitation in Charipinae hyperparasitoids (Hymenoptera: Figitidae) is notoriously difficult due to their minute size and limited morphological variability. Traditional diagnostic characters sometimes show intraspecific variation, raising concerns about their reliability. Here, we applied an integrative taxonomic framework to evaluate species boundaries among six species of *Alloxysta* Förster and four species of *Phaenoglyphis* Förster. We combined a morphological dataset of 53 characters with data from three molecular markers (COI, ITS2, and 16S rRNA) and reconstructed phylogenies under maximum-likelihood criteria. Phylogenies consistently recovered morphologically defined taxa as well-supported clades, confirming the overall reliability of traditional characters (pronotal and propodeal carinae, radial cell shape, and flagellomere proportions). On the other hand, molecular evidence refined certain species limits and highlighted cases of potential cryptic variation. Our results demonstrate that morphology still provides a strong baseline for Charipinae taxonomy, but integration with molecular data yields more robust and stable classifications. This study underscores the value of multi-locus approaches for resolving taxonomic ambiguities and provides a framework for future ecological and evolutionary research on these hyperparasitoid wasps.

## Simple Summary

Charipinae (Hymenoptera, Figitidae) are a small group of important aphid hyperparasitoids known for their tiny size and minimal visible variation, making them challenging to identify. Traditional morphological characters sometimes vary within species, making species delimitation unstable. We used an integrative taxonomic approach that combines morphology and molecular data to clarify species limits in two genera, *Alloxysta* and *Phaenoglyphis*. By analyzing 53 morphological characters and three genetic markers (COI, ITS2, and 16S rRNA), we found that molecular and morphological data largely agree, confirming the traditionally recognized species in this group. Furthermore, DNA evidence also helped refine some species boundaries and revealed possible hidden diversity. Our results show that morphology remains a reliable foundation for Charipinae taxonomy, but combining it with molecular data leads to more accurate and stable identification. This integrative framework will largely support future ecological and evolutionary studies of these hyperparasitoids.

## 1. Introduction

The Charipinae (Hymenoptera: Figitidae) are a cosmopolitan group of hyperparasitoid wasps that play an important role in aphid–parasitoid communities [1,2,3]. Eight genera are currently recognized, with distinct biogeographic distributions: *Alloxysta* Förster and *Phaenoglyphis* Förster are cosmopolitan, *Lytoxysta* Kieffer is restricted to North America, *Lobopterocharips* Paretas-Martínez & Pujade-Villar is confined to Nepal, *Dilyta* Förster is widespread except in Australia, *Apocharips* Fergusson spans the Eastern Palaearctic and Neotropical regions, *Dilapothor* Paretas-Martínez & Pujade-Villar and *Thoreauana* Girault are endemic to Australia [4,5]. Despite their bio-ecological significance, the taxonomy of Charipinae has historically been problematic [4]. Early species descriptions were vague and superficial, often based on coloration and sometimes lacking reference to the reliable morphological diagnostic characters, which led to a proliferation of synonyms and unstable classifications.

The uniform miniaturized sculpture, which differs slightly from the smooth body definition of Charipinae, further complicates identification, as many species show limited external variation and few clear diagnostic features [6]. Consequently, delimiting species boundaries remains a persistent challenge, particularly in the specious genera such as *Alloxysta* and *Phaenoglyphis*. Although recent revisions have brought greater clarity [3,5], unresolved ambiguities remain, especially among morphologically similar species. Traditional taxonomy of Charipinae has relied mainly on morphological characters such as the presence/absence and shape of pronotal and propodeal carinae, the size and shape of the radial cell, and the proportions of flagellomeres and rhinaria [3,6]. However, these characters often display high intraspecific variability, which can obscure interspecific differences and result in misidentifications [5]. Indeed, recent studies have shown that reliance on morphology alone is insufficient to resolve species boundaries, leading to the recognition of integrative approaches that combine morphology with molecular data [7,8,9,10].

DNA barcoding and phylogenetic analyses have been increasingly applied to the Charipinae, offering new perspectives on diversity and classification. For instance, different molecular markers were used by Ferrer-Suay et al. [8] to refine interspecific limits, with some species limits being confirmed [8] or a group of species being synonymized [7], while morphological and barcode data were integrated by Vogel et al. [10] to clarify species boundaries in *Phaenoglyphis*. Such studies highlight both the utility and the limitations of molecular markers, as divergence thresholds vary among species and single-locus approaches may produce misleading results [11]. Multi-locus datasets, combined with morphology, provide a more robust basis for species delimitation and have the potential to uncover cryptic diversity.

The aim of the present study is to evaluate the reliability of traditional morphological characters used in Charipinae taxonomy and to test their concordance with molecular evidence. We focus on six species of *Alloxysta* and four of *Phaenoglyphis*, integrating a comprehensive morphological dataset with three molecular markers (COI, ITS2, and 16S rRNA) under a phylogenetic approach. These species were selected based on the availability of specimens and because all three molecular markers could be obtained for them, allowing for a complete comparison and a more integrated analysis, by comparing genetic distances and phylogenetic groupings with morphology-based identifications, we assess the robustness of current species boundaries, detect potential cryptic taxa, and contribute to a more stable classification of Charipinae.

## 2. Materials and Methods

Collection and Identification

Specimens of Charipinae examined in this study (see Table 1) were collected in Yprès, Belgium, during the summer of 2022 as part of a separate research project (Verheyde et al., in prep.). Specifically, 80 specimens belonging to six species from the genera *Alloxysta* and four *Phaenoglyphis* belonging to a specific RBINS (Royal Belgian Institute of Natural Sciences) project were analyzed to assess the reliability of traditional morphological characters used in Charipinae taxonomy. Only specimens which were positive for the three molecular markers were selected for this analysis.

Morphological Characterization

Morphological assessments were conducted using a stereomicroscope (OPTIKA ZSM-2) and environmental scanning electron microscopes: an FEI Quanta 200 ESEM at the University of Barcelona and a Hitachi S4800 at the SCIE (Central Research Support Service) of the University of Valencia. Terminology for morphological traits follows Paretas-Martínez et al. [6]. Measured structures included flagellomeres F1–F12, the width of the forewing radial cell (from wing margin to the Rs vein), the transfacial line (distance across the face between compound eyes through the antennal sockets divided by eye height), and malar space (distance from the lower gena to the compound eye’s ventral margin, also normalized by eye height). A total of 53 morphological characters were selected for this study, extracted from Paretas-Martínez et al. [6], covering traits from various morphological regions (Table S1). First, specimens were morphologically identified using diagnostic characters and available taxonomic keys [12] to ensure accurate species-level determination. Subsequently, DNA was extracted from selected individuals—typically using non-destructive methods to preserve voucher specimens—so that molecular analyses could complement and validate the morphological identifications.

DNA Extraction, Amplification, and Sequencing

The methodological approach applied in this work follows the protocol described in Ferrer-Suay et al. [8], as the analytical workflow fully matches that of the previously published work. We therefore implemented the same procedures, including molecular processing using established DNA extraction and sequencing methods, and subsequent integrative analyses.

Phylogenetic analyses

The phylogenetic procedures applied in this study follow the same analytical framework described in Ferrer-Suay et al. [8], as the structure and requirements of the dataset are equivalent. As in the previously published work, we included *Anacharis zealandica* Ashmead, 1900 (Hymenoptera: Figitidae: Anacharitinae) as the outgroup and used three molecular markers (partial COI, 16S, and ITS2). Sequence alignment, quality control through amino acid translation, and subsequent dataset preparation were conducted following the workflow established in that previous study.

Similarly, we compiled a morphological matrix comprising all informative characters and performed Maximum Likelihood analyses on both individual and concatenated datasets using the same partitioning strategy, substitution model selection approach, and ML settings as previously reported. Model selection, partition optimization, and nodal support estimation (including ultrafast bootstrap procedures) thus fully replicate the methodology of Ferrer-Suay et al. [8]. All phylogenetic reconstructions and distance analyses (e.g., K2P estimates) were carried out using the same software suite and analytical criteria detailed therein.

## 3. Results

**Figure 1:**
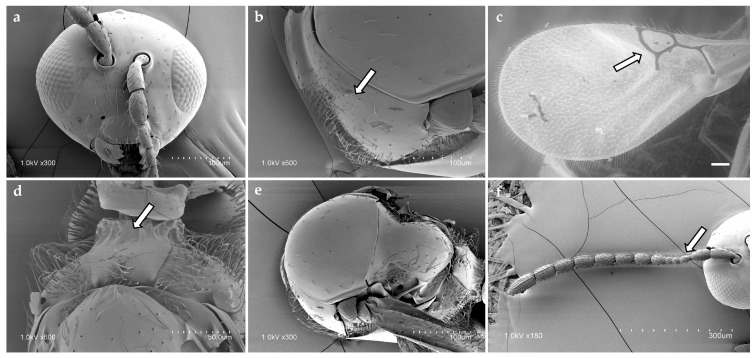
Diagnostic morphological features of *Alloxysta brevis* (Thomson, 1862): (**a**) head, (**b**) pronotum, arrow shows absence of pronotal carinae; (**c**) fore wing, arrow shows radial cell, (**d**) propodeum, arrow shows propodeal plate; (**e**) mesoscutum; (**f**) antenna, arrow shows F1–F3.

**Figure 2:**
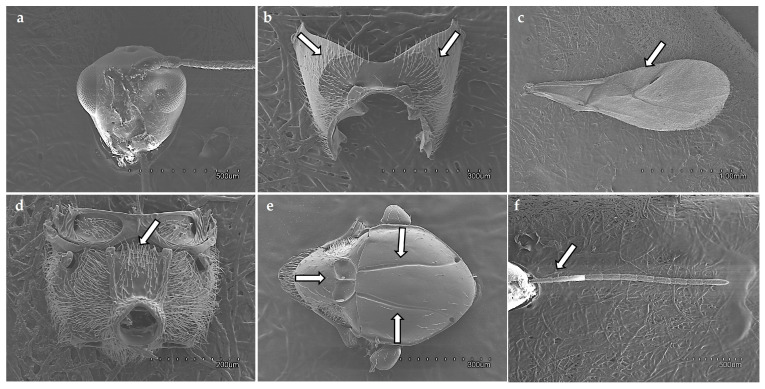
Diagnostic morphological features of *Phaenoglyphis xanthochroa* Förster, 1869: (**a**) head, (**b**) pronotum, arrows show pronotal carinae; (**c**) fore wing, arrow shows radial cell, (**d**) propodeum, arrow shows propodeal carinae; (**e**) mesoscutum, arrows show notauli and scutellar foveae; (**f**) antenna, arrow shows F1–F3.

17 Charipinae species were identified. However, due to concordance of different sequences, only ten were included in the final study (Table 1): *A. brevis* (Thomson, 1962) (Figure 1), *A. castanea* (Hartig, 1841), *A. obscurata* (Hartig, 1840), *A. pilipennis* (Hartig, 1840), *A. ramulifera* (Thomson, 1862), *A. victrix* (Westwood, 1833), *P. heterocera* (Hartig, 1841), *P. longicornis* (Hartig, 1840), *P. villosa* (Hartig, 1841), and *P. xanthochroa* Förster, 1869 (Figure 2). Material from *Alloxysta arcuata* (Kieffer, 1902), *Alloxysta halterata* (Thomson, 1862) and *Alloxysta citripes* (Thomson, 1862) was very old and it was not possible to extract molecular information. *Alloxysta consobrina* (Zetterstedt, 1838) was recently synonymized with *A. victrix* (Ferrer-Suay et al., [7]). From the *Phaenoglyphis* genus, *P. belizini* Pujade-Villar, 2019, *P. nigripes* (Thomson, 1862) and two doubtful species are included in a genus-level revision (Barreda-Llorens et al., in prep.). In total, 153 sequences were obtained for this project (CHARIPIMOL CIGE/2022/158): COI:60, ITS2: 53 and 16S: 40.

**Figure 3:**
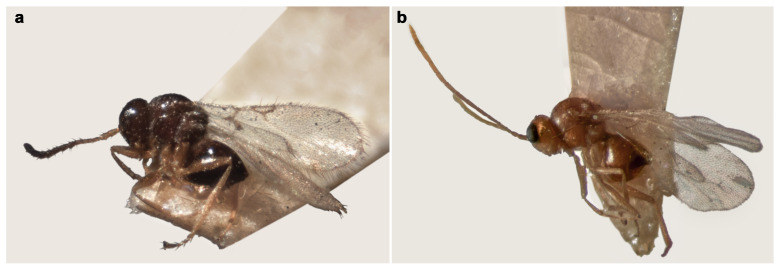
(**a**) Habitus of *Alloxysa brevis* (Thomson, 1862), type deposited at Lund Museum of Zoology (Sweden); (**b**) habitus of *Phaenoglyphis xanthochroa* Förster, 1869, type deposited at Zoologisches Museum Humboldt-Universität (Germany).

Diagnostic characters of the radial cell, pronotal and propodeal sculpturing, and flagellar proportions were used to assign individuals to species (Table 2). *Alloxysta brevis* (Thomson, 1862) is characterized by a small and closed radial cell, absence of pronotal carinae, presence of a propodeal plate, and short flagellomeres F1–F3, which are subequal in length. *Alloxysta castanea* (Hartig, 1841) shows a partially open radial cell, distinct carinae on both the pronotum and propodeum, and a clavate F3. *Alloxysta obscurata* (Hartig, 1840) has a partially open radial cell, with carinae present on the pronotum and propodeum. *Alloxysta pilipennis* (Hartig, 1840) is distinguished by a closed radial cell, well-developed carinae on the pronotum, a propodeal plate, and an F1 longer than the pedicel. *Alloxysta ramulifera* (Thomson, 1862) possesses a closed radial cell, a propodeal plate, and small pronotal carinae. *Alloxysta victrix* (Westwood, 1833) exhibits a large and closed radial cell, distinct pronotal carinae, and lacks a propodeal plate. Within *Phaenoglyphis*, *P. heterocera* (Hartig, 1841) is recognized by the clavate shape of F3; *P. longicornis* (Hartig, 1840) by its elongated F1, which is distinctly longer than the remaining flagellomeres and clavate in shape; and *P. villosa* (Hartig, 1841) by a partially open radial cell and absence of notauli. All diagnostic features were compared with type material, descriptions and reference specimens to confirm species identification. In addition to the molecular results, the study involved a thorough analysis of the key morphological characteristics that have traditionally been used in Charipinae taxonomy. These traits include the presence or absence of pronotal (PN) and propodeal (PP) carinae, the shape and size of the radial cell (RC), the proportions of the flagellomeres (FG), the onset of rhinaria and the morphology of the clava (RN), as summarized in Table 2. Combining the analysis of these characters with molecular data allowed for a more comprehensive assessment of interspecific variation and diagnostic reliability. Figure 3 shows the habitus of example specimens representing each studied genus.

The analysis of genetic distances based on COI revealed a wide spectrum of evolutionary divergence within the dataset (Table S2), ranging from identical sequences (0.000) between conspecific individuals of *A. victrix*, *A. brevis*, *A. pilipennis*, and *P. longicornis*, indicating strong intraspecific conservation, to substantial interspecific difference. The smallest interspecific distance (0.0626) was observed between specimens of *A. ramulifera* and *A. pilipennis*, suggesting a very recent divergence or minor variation within the species. In contrast, the largest distances highlighted deep evolutionary splits, particularly involving *A. obscurata* with *A. brevis* (0.1342).

**Figure 4:**
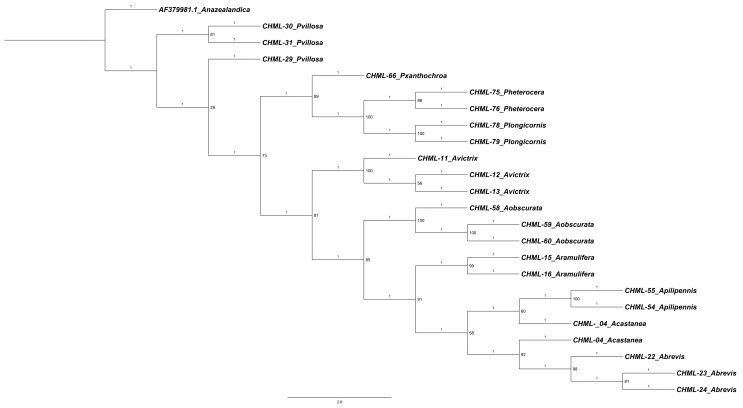
Maximum likelihood tree inferred with IQ-TREE v3 under the best-fit substitution model automatically selected for each partition. The analysis was performed in safe mode with 1000 ultrafast bootstrap (UFBoot) replicates. Numbers on nodes indicate UFBoot support values (%) and on branches the specific branch length (raw).

The phylogenetic tree obtained (Figure 4) reveals a well-resolved topology that corroborates the patterns of genetic distancing previously calculated. Several well-supported clades are observed, grouping individuals of the same species, consistent with the minimum genetic distances (0.000–0.0052) recorded in the matrix. Relationships with greater divergence are also reflected in the topology of the tree. The sequence AF379981.1 (*Anacharis zealandica*), which had the largest distances (up to 0.7773) with all other sequences, is positioned as the most basal and divergent lineage in the tree, confirming its status as a clearly differentiated outgroup. Among the *Alloxysta* taxa, the tree shows a clear separation between most of the species, forming distinct, non-sister clades. It is worth noting that *A. castanea* (CHML-04) appears duplicated in the tree, suggesting possible redundancy in the analysis or a high degree of genetic conservation with other taxa. The genus *Phaenoglyphis* (*P. villosa*, *P. xanthochroa*, *P. longicornis*, *P. heterocera*) forms a coherent group distinct from *Alloxysta*, supported by consistently larger genetic distances between genera than within them; however, our results here show it as a paraphyletic group.

## 4. Discussion

Our results demonstrate that integrative taxonomy provides a robust framework for delimiting species of Charipinae. Phylogenetic analyses based on three independent markers (COI, ITS2, 16S) consistently recovered clades that correspond to morphologically defined species, confirming the reliability of classical characters such as pronotal and propodeal carinae, radial cell shape, and flagellomere proportions.

Comparison with previous studies revealed both stable and variable divergence patterns within Charipinae. Ferrer-Suay et al. [8] reported interspecific COI distances of 12.4–16.4%, which are higher than those found in our study (0.6–12.8%). Nevertheless, both studies converge in demonstrating that molecular data generally corroborate morphology-based delimitations while refining problematic boundaries. Similar findings have been reported in *Phaenoglyphis*, where barcode data have revealed unexpected phylogenetic placements, such as the nesting of *P. villosa* within *Alloxysta* [8], however this genus has a 6 bp long deletion in the CO1 barcode region unique amongst Figitidae [9]. These cases illustrate the limitations of morphology alone and the importance of integrative approaches for stabilizing Charipinae taxonomy. The paraphyly of *Phaenoglyphis* is currently being examined as part of an extensive taxonomic revision of the genus aimed at clarifying its phylogenetic boundaries. Similarly, the intraspecific variability observed in *Alloxysta castanea* is the subject of an ongoing revisionary study intended to reassess its diagnostic characters and species limits.

Beyond Charipinae, our results align with broader trends in Hymenoptera systematics. The performance of ITS2 as a marker, for instance, has been questioned in other parasitoid groups due to intragenomic variation [10]), while standardized divergence thresholds such as the 2% COI cutoff have proven unreliable across insects [11]. Our study avoids these pitfalls by combining multiple loci with morphological data, thereby providing a taxon-specific framework for species delimitation. The clear genetic gap recovered here contrasts with more gradual divergence patterns reported in other parasitoids, such as *Lariophagus distinguendus* (Pteromalidae, Förster, 1841), where reproductive isolation accumulates more progressively [12].

Despite these advances, several limitations remain. Our dataset is geographically restricted, and broader sampling will be essential to assess potential cryptic diversity and refine species limits across regions. In particular, the unusually high intraspecific variability detected in *A. obscurata* warrants further investigation, as well as resolving the *Alloxysta castanea* complex. Future research should also incorporate genome-wide markers (e.g., target enrichment, RADseq) to achieve higher resolution in species boundaries and evolutionary relationships. Finally, integrating host–parasitoid interaction data with molecular analyses could provide a more complete picture of the ecological roles and evolutionary dynamics of Charipinae. In conclusion, this study confirms that traditional morphological traits remain a reliable foundation for Charipinae taxonomy, but their integration with molecular evidence yields more accurate and stable classifications. By establishing a robust framework for species delimitation, our results contribute to the systematic, ecological, and applied understanding of this ecologically significant group of hyperparasitoid wasps.

## 5. Conclusions

This study shows that integrative taxonomy—combining morphology with multilocus molecular data—provides a robust framework for delimiting Charipinae species. Morphological traits remain reliable, but molecular evidence helps resolve problematic boundaries, as seen in the *Phaenoglyphis* paraphyly and the *Alloxysta castanea* complex. Although our results generally align with previous studies, geographic expansion of sampling and the use of genome-wide markers will be essential to refine species limits and detect potential cryptic diversity. Overall, integrating morphological and molecular approaches yields more accurate and stable classifications, improving our understanding of the diversity and evolutionary relationships within Charipinae.

## Figures and Tables

**Table 1:** List of Charipinae samples analyzed in this study. The reference code (CHML-XX), the assigned species and availability of molecular sequences for the three markers used (COI, ITS2 and 16S) are indicated. The “MOUNTED” column specifies the assembly status of the copies.

Internal	Code	Genus	Species	Genbank Accession Number	Reference
CHML_04	1753	*Alloxysta*	*castanea*	16S: PP109087 COI: PP097396 ITS2: PP102679	Ferrer-Suay et al., [8]
CHML_11	1752	*Alloxysta*	*victrix*	16S: SUB15768782 COI: PP097406 ITS2: SUB15768782	Ferrer-Suay et al., [8]
CHML_12	1752	*Alloxysta*	*victrix*	16S: SUB15768782 COI: PP097407 ITS2: SUB15768782	
CHML_13	1752	*Alloxysta*	*victrix*	16S: SUB15768782 COI: PP097408 ITS2: SUB15768782	
CHML_15	1749	*Alloxysta*	*ramulifera*	COI: PX677500 ITS2: PX667120	This study
CHML_16	1749	*Alloxysta*	*ramulifera*	COI: PX677501 ITS2: PX667121	
CHML_22	1755	*Alloxysta*	*brevis*	16S: PX664103 COI: PX677502 ITS2: PX667122	
CHML_23	1755	*Alloxysta*	*brevis*	16S: PX664104 COI: PX677503 ITS2: PX667123	
CHML_24	1755	*Alloxysta*	*brevis*	16S: PX664105 COI: PX677504 ITS2: PX667124	
CHML_29	1753	*Phaenoglyphis*	*villosa*	16S: PP109110 COI: PP097430 ITS2: PP102706	Ferrer-Suay et al., [8]
CHML_30	1753	*Phaenoglyphis*	*villosa*	16S: PX664107 COI: PX677506 ITS2: PX667126	
CHML_31	1753	*Phaenoglyphis*	*villosa*	16S: PX664108 COI: PX677507 ITS2: PX667127	
CHML_54	1751	*Alloxysta*	*pilipennis*	16S: PX664109 COI: PX677508 ITS2: PX667128	This study
CHML_55	1751	*Alloxysta*	*pilipennis*	16S: PX664110 COI: PX677509 ITS2: PX667129	
CHML_58	1805	*Alloxysta*	*obscurata*	COI: PX677510 ITS2: PX667130	
CHML_59	1749	*Alloxysta*	*obscurata*	COI: PX677511 ITS2: PX667131	
CHML_60	1752	*Alloxysta*	*obscurata*	COI: PX677512 ITS2: PX667132	
CHML_66	1815	*Phaenoglyphis*	*xanthochroa*	COI: PX677513 ITS2: PX667133	
CHML_75	1748	*Phaenoglyphis*	*heterocera*	16S: PX664111 COI: PX677514 ITS2: PX667134	
CHML_76	1746	*Phaenoglyphis*	*heterocera*	16S: PX664112 COI: PX677515 ITS2: PX667135	
CHML_78	1746	*Phaenoglyphis*	*longicornis*	16S: PX664113 COI: PX677516	
CHML_79	1746	*Phaenoglyphis*	*longicornis*	16S: PX664114 COI: PX677517	

**Table 2:** Comparative diagnostic characteristics for the Charipinae species analyzed in this study. PN—presence/absence and shape of pronotal carinae; PP—and propodeal carinae; RC—radial cell size and shape; FG—flagellomere proportions.

Charipinae Species	PN	PP	RC	RN	FG
*A. brevis*	−	+	closed	F4	F1–F3 < F4
*A. castanea*	+	+	parc. open	F3	F2–F4 subequal
*A. obscurata*	+	−	parc. open	F4	F1 > F2, F2 < F3, F3 < F4
*A. pilipennis*	+	+	closed	F3	F2–F4 subequal
*A. ramulifera*	+	+	closed	F4	F1 > F2 = F3
*A. victrix*	+	−	closed	F3	F1 > F2, F2–F4
*P. heterocera*	+	+	closed	F3	F1 > F2 < F3 = F4
*P. longicornis*	+	+	closed	F1	F1 > F2 = F3 < F4
*P. villosa*	+	+	parc. open	F3	F1 > F2–F4
*P. xanthochroa*	+	+	closed	F3	F1 > F2 < F3 = F4

## Data Availability

The original contributions presented in this study are included in the article. Further inquiries can be directed to the corresponding author(s).

## Supplementary Materials

The following supporting information can be downloaded at: https://www.mdpi.com/article/10.3390/insects17010054/s1.

## References

[1] Muller C.B., Adriaanse I.C.T., Belshaw R., Godfray H.C.J. (1999). The structure of an aphid–parasitoid community. J. Anim. Ecol..

[2] Mkenda P.A., Ndakidemi P.A., Stevenson P.C., Arnold S.E.J., Belmain S.R., Chidege M., Gurr G.M., Woolley V.C. (2019). Characterization of hymenopteran parasitoids of Aphis fabae in an African smallholder bean farming system through sequencing of COI ‘Mini-barcodes’. Insects.

[3] Ferrer-Suay M., Selfa J., Pujade-Villar J. (2021). A review of the subfamily Charipinae (Hymenoptera: Cynipoidea: Figitidae): Hyperparasitoids potentially affecting the biological control of aphids. Ann. Soc. Entomol. Fr..

[4] Ferrer-Suay M., Paretas-Martínez J., Selfa J., Pujade-Villar J. (2012). Taxonomic and synonymic world catalogue of the Charipinae and notes about this subfamily (Hymenoptera: Cynipoidea: Figitidae). Zootaxa.

[5] Ferrer-Suay M., Selfa J., Pujade-Villar J. (2023). Overview of the current status of Charipinae (Hymenoptera: Cynipoidea: Figitidae): Taxonomy, distribution, and diversity. Zootaxa.

[6] Paretas-Martínez J., Arnedo M.A., Melika G., Selfa J., Seco-Fernández M.V., Fülöp D., Pujade-Villar J. (2007). Phylogeny of the parasitic wasp subfamily Charipinae (Hymenoptera: Cynipoidea: Figitidae). Zool. Scr..

[7] Ferrer-Suay M., Cuesta-Porta V., Selfa J., Pujade-Villar J. (2025). Clarification of the taxonomic status of two *Alloxysta* species through genetic analysis (Figitidae: Charipinae). Diversity.

[8] Ferrer-Suay M., Bulgarella M., Heimpel G.E., Rakhshani E., Selfa J. (2024). Interspecific limits within Charipinae (Cynipoidea: Figitidae), insights from molecular data. Insects.

[9] Vogel J., Peters R.S., Selfa J., Ferrer-Suay M. (2024). Characterising the north-western European species of *Phaenoglyphis* Förster, 1869 (Hymenoptera: Figitidae: Charipinae) with novel insights from DNA barcode data. Biodivers. Data J..

[10] Van Veen F.F., Belshaw R., Godfray H.C.J. (2003). The value of the ITS2 region for the identification of species boundaries between *Alloxysta* hyperparasitoids (Hymenoptera: Charipidae) of aphids. Eur. J. Entomol..

[11] Cognato A.I. (2006). Standard percent DNA sequence difference for insects does not predict species boundaries. J. Econ. Entomol..

[12] Pollmann M., Kuhn D., König C., Homolka I., Paschke S., Reinisch R., Schmidt A., Schwabe N., Weber J., Gottlieb Y. (2023). New species based on the biological species concept within the complex of *Lariophagus distinguendus* (Hymenoptera, Chalcidoidea, Pteromalidae), a parasitoid of household pests. Ecol. Evol..

