# Supplementary material for: Deciphering Morphological Variability: Addressing Taxonomic Ambiguities in Contemporary Species Delimitation (Hymenoptera, Figitidae)
Source: Insects. 2026 Jan 1;17(1):54. doi: 10.3390/insects17010054 (PMC12842034; doi:10.3390/insects17010054)

0. Shape of frons: **(0)** straight; **(1)** convex.
1. Frontal ocellus: **(0)** almost aligned with lateral ocelli; **(1)** forming a triangle with lateral ocelli.

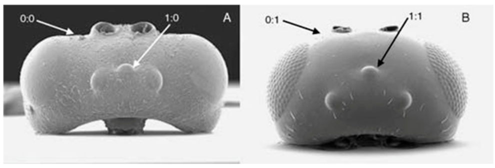

2. Clypeus: **(0)** not projecting over mandibles; **(1)** slightly projecting over mandibles; **(2)** broadly projecting over mandibles.

3. Epistomal sulcus: **(0)** present, at least marked by a distinct change of curvature at face; **(1)** absent.

4. Distance between tentorial pits: **(0)** shorter than distance from tentorial pits to clypeal margin; **(1)** longer.

5. Clypeo-pleurostomal lines: **(0)** present, at least marked by a change of curvature at lower face; **(1)** absent.

6. Facial strigae: **(0)** present, radiating from clypeus; **(1)** absent.

7. Malar impression: **(0)** absent; **(1)** present.

8. Transfacial line: **(0)** 1-1,25 times eye's height; **(1)** < eye's height; **(2)** > 1,75 eye's height.

9. Shape of dorsal margin of the head: **(0)** straight; **(1)** convex.

10. Number of ommatidia in a distance equal to toruli width: **(0)** around 10; **(1)** 4-6.
11. Face setae: **(0)** abundant, homogeneously distributed on face and frons; **(1)** sparse, concentrated below the toruli.

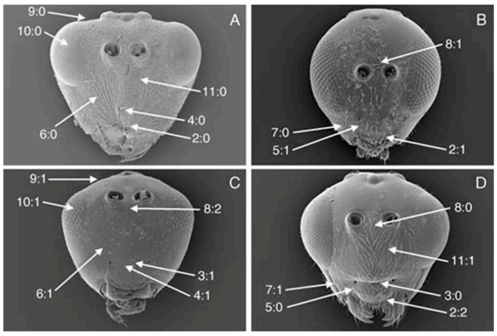

12. Number of flagellomeres (female): (0) 12; (1) 11; (2) 10; (3) 9.

13. Apical club in female antenna: (0) absent; (1) present, formed by 2 segments; (2) present but unsegmented.

14. Pedicel (female): (0) as long as wide; (1) longer than wide but smaller than F1; (2) globular, bigger than F1; (3) cup-shaped.

15. Relation between F1 and F2: (0) F1 longer than F2; (1) F1 subequal to F2; (2) F1 shorter than F2.
16. Relation between F2 and F3: (0) F2 longer than F3; (1) F2 subequal to F3; (2) F2 shorter than F3.
17. Beginning of rhinaria and club shaped: (0) F2; (1) F3; (2) F4.

18. Shape of F1 (male): (0) without modifications; (1) excavated or curved; (2) with a prominent hump.

19. Shape of F2 (male): (0) without modifications; (1) excavated or curved.

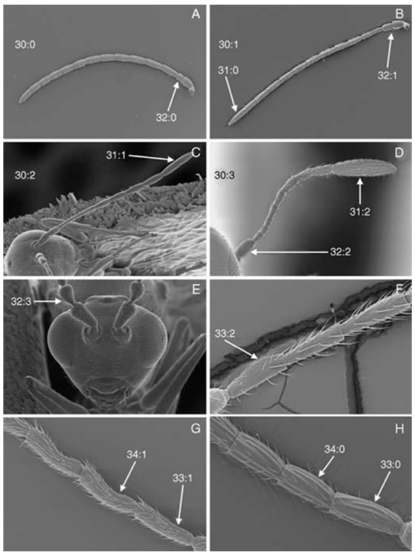

20. Pronotal carinae: (0) absent; (1) short; (2) long; (3) very large, forming a well upraised pronotal plate.

21. Anterior part of subpronotal plate: (0) with two folds separated by a flat surface; (1) wrinkled, with two folds; (2) curved.

22. Processes behind submedian pronotal depression: (0) big, defining a subpronotal plate; (1) small, punctual; (2) absent.

23. Pronotum setosity: (0) abundant on all the pronotum; (1) abundant anteriorly, glabrous or sparse posteriorly.

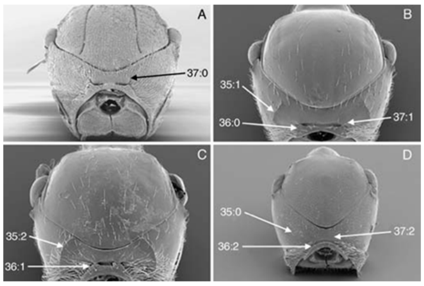

24. Mesopleuron surface: (0) with wrinkles and/or carinae; (1) with a single sulcus; (2) smooth, plain.
25. Mesopleural triangle: (0) present; (1) absent.

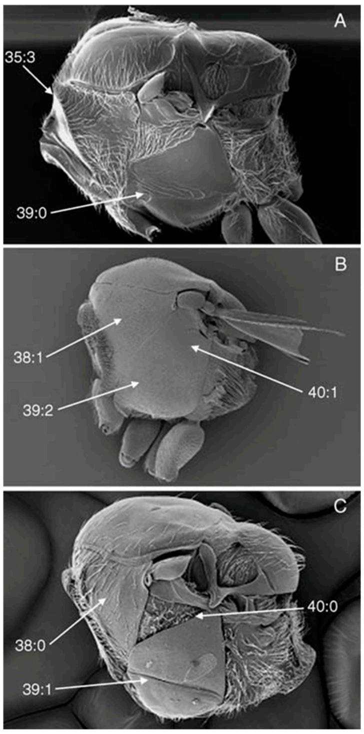

26. Scutum setosity: (0) abundant; (1) sparse glabrous.

27. Anterior admedian signum: (0) present, at least by two strips without setae; (1) absent.

28. Imbricated sculpture in the scutum: (0) on the entire scutum; (1) only on small areas next to notauli; (2) absent.

29. Notauli: (0) deep, well-marked; (1) superficial, only slightly marked; (2) absent.

30. Median mesoscutal impression: (0) present; (1) absent.

31. Parascutal carina: (0) anteriorly ending next to tegula; (1) continuing reaching pronotal carinae.
32. Scutellar foveae: (0) present; (1) absent.

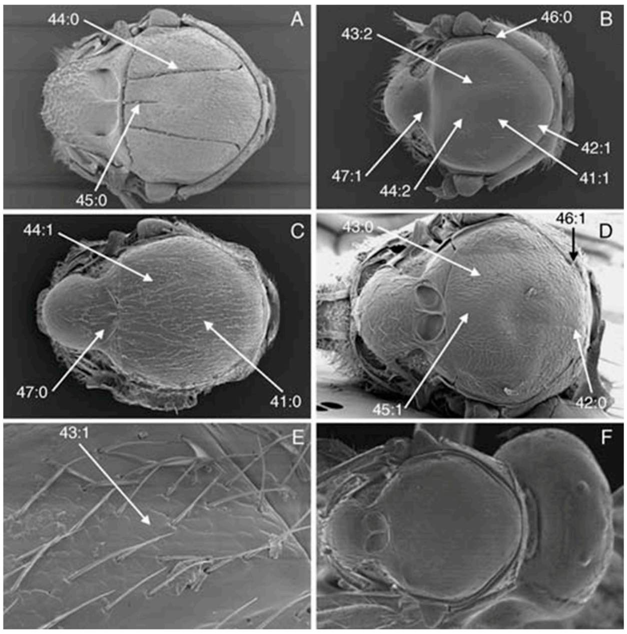

33. Subaxillar bar: (0) posterior end with a dorsal projection; (1) continuous with posterior part of scutellum.
34. 01 carinae; (1) with carinae; (2) symmetric carinae at both sides; (3) with a projected plate.
35. Metascutellum: (0) distinctly constricted medially; (1) subrectangular.
36. Sculpture on metascutellum: (0) one longitudinal medial carina; (1) strongly sculptured; (2) with only some wrinkles.
37. Sculpture on metanotal trough ventral bar: (0) present; (1) absent.
38. Metanotal trough: (0) clearly marked; (1) not delimitated on its inferior margin.
39. Propodeum carinae: (0) present; (1) absent; (2) the entire propodeum with irregular carinae.
40. Shape of propodeum carinae: (0) narrow; (1) broad; (2) forming a propodeal plate; (3) incomplete.
41. Medial acetabular carina: (0) long; (1) very short.

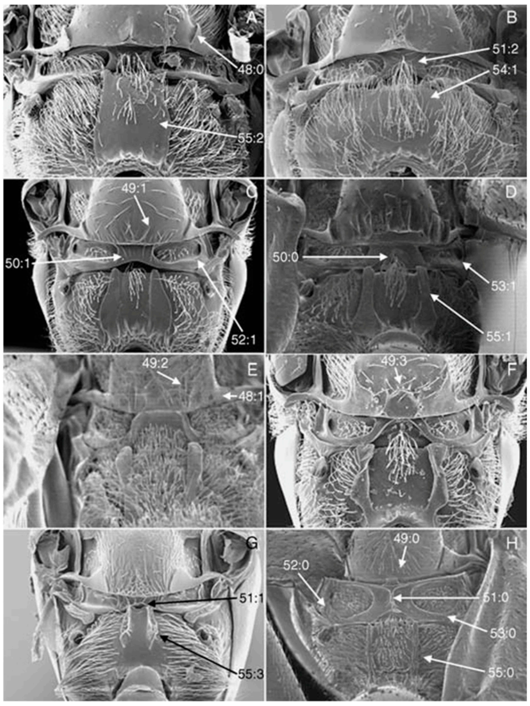

42. Shape of wing: (0) asymmetrical; (1) almost symmetrical; (2) with a ondulation in its apical ventral part.
43. R1vein: (0) complete; (1) not reaching Rs; (2) reaching anterior margin; (3) not reaching anterior margin.
44. Rs vein: (0) complete; (1) incomplete.
45. Shape of Rs: (0) not parallel to R1; (1) parallel to R1.
46. 2rm vein: (0) clearly indicated; (1) only marked by a small beginning; (2) very thick.

47. Veins aspect: (0) hollowed; (1) not hollowed.

48. Rs+M vein: (0) pointing to middle of basal vein; (1) pointing to posterior end of basal vein; (2) absent.

49. M, Cu1a and M+Cu1a veins: (0) present; (1) absent.

50. Marginal setae: (0) present; (1) absent.

51. T3-T4: (0) dorsomedian lengths subequal; (1) T3 very small; (2) fused.

52. Ring of setae at base of T3: (0) absent, only a few sparse setae present; (1) present.

53. Apex of ventral spine: (0) projected; (1) not projected.

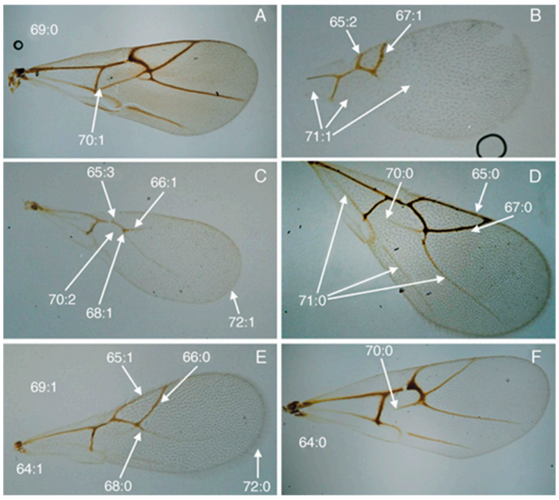

Supplement: Supplementary file 1 [file insects-17-00054-s001.zip › Morphological features_Supplementary 1.pdf]
